# The design of the arrangement of evacuation routes on a passenger ship using the method of genetic algorithms

**DOI:** 10.1371/journal.pone.0255993

**Published:** 2021-08-09

**Authors:** Dorota Łozowicka

**Affiliations:** Maritime University of Szczecin, Szczecin, Poland; Torrens University, AUSTRALIA

## Abstract

The article concerns the problem of evacuation from passenger ships. It is important because it has not yet been possible to eliminate all the hazards associated with sea travel. In this paper, a concept of a method allowing to determine the arrangement of evacuation routes, for which evacuation time would be minimal, was presented. The genetic algorithm method was used in the calculations, and an original method of coding the considered problem was proposed. Sample calculations were performed to verify the correctness of the proposed algorithm. The results of applying the developed method to calculate the evacuation time on a real passenger ship are presented.

## Introduction

The development of the shipbuilding industry in the sector related to the construction of modern passenger ships is characterized in recent years by the trend to build larger and larger vessels, taking on board up to five thousand people. A passenger ship has to be designed and operated to be safe, but there are still accidents that make it necessary to evacuate passengers and crew from the ship.

At the ship design stage and during the development of evacuation procedures, the question arises, how should the evacuation routes on the ship be designed to reduce the evacuation time as much as possible? It is necessary to improve the "survivability" of the ship, but it is also important to develop evacuation systems in terms of rescue operations as well as the design of evacuation routes on the ship.

Evacuation routes should have adequate capacity, signage and lighting. The time it takes for passengers to get from their evacuation areas to their assembly stations is highly dependent on human factors. Most passengers are usually unfamiliar with the layout of corridors and various spaces on the ship, which can cause difficulties in locating assembly areas. It is therefore important that assembly stations are easily identifiable and the routes leading to them clearly signed. Reduced evacuation time is influenced by, among other things, the correct arrangement of escape routes. Due to the impossibility of influencing the randomness of the population among passengers (e.g. the presence of intoxicated people, invalids, etc.), evacuation routes should primarily be designed in such a way that the evacuation time is as short as possible.

The following reasons should be considered when determining the purpose of the study:

- the construction of large passenger ships carrying thousands of people generates the problem of safe evacuation of these people in case of such necessity;
- it is not always possible to eliminate human errors leading to unsafe navigation at the stage of ship design, construction and operation, therefore it should be assumed that evacuation from a passenger ship should be possible;
- there is a need to shorten the evacuation time, including moving people from the places where they are alerted to the assembly stations.

The purpose of this paper is to present the concept of a method to determine the arrangements of evacuation routes for which evacuation time would be minimal.

The ship design philosophy focuses primarily on improving the survivability of ships—new passenger ships must be designed so that they can safely return to port in case of fire or flooding of a watertight compartment [1–3]. In the case of an accident, systems such as propulsion, steering, navigation and fuel systems must be operational so that the ship is able to return to port. It is important to maintain communications and fire prevention systems. The problem of a ship’s safe return to port in the case of a fire can be found in the publication [4]. The method of quantitative analysis was presented to solve the problem of uncertainty of the evacuation time of passengers.

One of the most dangerous causes of evacuation is fire. The growth of a fire is influenced, among others, by the accumulation of combustible materials and their type, air supply and outflow of combustion products, temperature. Fire products are dangerous for the evacuation process. As a result of the combustion process, thermal energy is released and fire gases, smoke, soot and ash are produced. These factors make it difficult or even impossible for evacuees to evacuate. Particularly in conditions of reduced visibility, it is common for people to lose their self-control, resulting in a panicked escape [5,6].

The publication [7] presents a three deck passenger ship and considers four different fire scenarios that cover typical fire locations. The risk model for passenger ship fire safety assessment at the design stage is presented in the publication [8]. In [9] a topological model of evacuation routes is established taking under consideration various fire scenarios.

The efficiency of the evacuation process is greatly influenced by the speed of people moving along the evacuation routes. It is influenced by the "human factor" and the environment in which the evacuation takes place, taking into account the difficulties that may delay the movement of people.

The publication [10] presents an evacuation simulation model based on agents for predicting crowd behavior and assessing the safety of evacuation on passenger ships. It is noted that there are passengers who are not familiar with the arrangement of the vessel and passengers who have family members or friends.

An agent based model was also presented in [11], however, the effect of obstacles in the cabins on the evacuation of passengers was considered. An important factor influencing the speed of people during evacuation is the presence of inclined surfaces on the ship.

The publications [12,13] analyzed the correlation between passenger localization and walking speed in the aspects of inclination. The publication [14] presents the current state of ship passenger safety awareness, perceptions of emergency wayfinding tools, and demographic differences in safety awareness and perceptions.

The genetic algorithm method has often been used to solve problems related to the safety and evacuation of people from both ships and land-based buildings. Planning evacuation from endangered areas (e.g. cities) in the event of an earthquake, flood, hurricane etc. using multi-criteria optimization by evolutionary algorithms is presented in the publication [15]. Similarly in the publication [16] the authors used a genetic algorithm optimization approach to improve the evacuation efficiency of a building complex, but also performed calculations for a twelve-deck cruise ship.

In the publication [17], attention is drawn to the problem of bottlenecked passageways (e.g. doors) which are a danger zone during evacuation. These are places endangered by the appearance of crowding and the threat of panic. The authors suggest limiting the flow of people to the emergency exit by placing obstacles on the evacuation route intentionally. Evolutionary algorithm methods were used to optimize the flow of people through emergency exits.

Evolutionary algorithms have been used to design urban spatial planning of streets, stores, shopping centers based on people’s decision making process in shopping planning [18]. This publication considers the flow of people in conditions without an emergency or evacuation necessity. However, it is a very interesting example of the universality of using the method of evolutionary algorithms.

In the publication [19] genetic algorithms were used to select the best variants of evacuation of people located in specific sectors on passenger quays. However, the publication [20] presents an evacuation plan generator using a genetic algorithm to reduce the total evacuation time.

The above-described applications of genetic algorithms for solving problems related to the evacuation and behavior of crowds, as well as the results of earlier studies prompted the author to use this method to solve the problem. Also, the publication [21] presents the method for solving optimization problems with opposing objectives and constraints in the decision-making process. Such problems are difficult to solve by traditional methods, so the methods of genetic algorithms are indicated. Such problems are difficult to solve with traditional methods, therefore the methods of genetic algorithms have been indicated.

The literature review should analyze the existing methods of describing the arrangement of evacuation routes on passenger ships in models simulating the process of evacuation. In all models it is necessary to present the environment in which the evacuation takes place, i.e. interior geometry (distribution of corridors, cabins).

As a general rule, each analyzed space is divided into subspaces, and each subspace is connected to its neighbors. Typically, the following methods are used to represent the environment:

- the space is covered with regular tiles of various sizes and shapes depending on the model (e.g. EGRESS model [22])
- each tile represents a room or corridor and does not match the actual dimensions; the models divide the building plan into rooms, corridors, stairs, etc., people move from one room to another (e.g. EXIT89 model [23])
- the rooms are presented as a continuous two-dimensional grid—models of this type impose a two-dimensional space on the building room diagram, while allowing people to move from one point of space to the next, it is possible to simulate the presence of obstacles and barriers inside the building, which directly affects the choice of escape route; the distance map is also plotted on the evacuation plan to map the distance from each cell of the analyzed structure to all exits and is generated using a series of recursive algorithms to determine the direct distance to the exit from any point on the plan (e.g. GridFlow model [24])

The first and third solutions allow the exact location of the person in the room and to identify obstacles and barriers. The second solution only allows you to analyze the movement of people between rooms

Each evacuation simulation model should have a specific application, i.e., it should be determined for which type of structure it can be used by a potential user. When applying results obtained for land buildings to ship applications, specific conditions on ships should be taken into account—movement of people may be restricted by the presence of inclined and unstable platforms, which is caused by ship motion. Models that can be used to analyze evacuation from most civic facilities, as well as ships, include EXODUS [25], ANEAS [26], EGRESS [22]. These models are complemented by modules for use in the marine industry.

The formulation and development of evacuation models can significantly improve the safety of life at sea. With model-based evacuation studies from passenger ships, many improvements in the design of evacuation routes can be made at the ship design stage, due to the possibility of a practically unlimited number of computer simulations. Evacuation models are thus used to verify the layout of evacuation routes. By analyzing the evacuation routes, bottlenecks can be identified at the design stage, i.e., the need for alternative evacuation route design can be identified.

However, evacuation models do not optimize the arrangement of evacuation routes so as to achieve the shortest evacuation time. They can only be used for possible evaluation of existing or planned solutions. Independent of the evacuation time calculation method used, it is assumed which routes people take to the assembly stations. The most appropriate routes (in the sense of time) are not identified. Many models assume that people follow the predetermined evacuation procedure. However, disruptions may occur during the actual evacuation, due, for example, to "herd behavior" tendencies, counter flow and panic, or other factors [27].

Therefore, the aim of the article is to fill the gap in research consisting in combining the modeling of the evacuation process with the optimization of the arrangement of evacuation routes in order to achieve the shortest possible evacuation time. Realizing the purpose of the research, it is possible to use some of the methods used in existing evacuation models concerning the method of coding the geometry of evacuation routes.

## Materials and methods

### Concept of a method to evaluate the validity of the design of evacuation routes

At the stage of ship design, the arrangement of evacuation routes can be verified, i.e. the dimensions of corridors, size and arrangement of staircases, arrangement of assembly stations, so as to obtain the shortest evacuation time. The problem of the evacuation process can be simplified to the transportation task, i.e. such an arrangement of evacuation routes for particular groups of people to obtain the shortest possible evacuation time. Based on the ship’s general arrangement, which includes the arrangement of evacuation routes, an evacuation organization plan can be created. This is a diagram of how passengers will be distributed from their locations (with the initial location determined for a specified evacuation scenario) to assembly stations (possibly safe areas). For the calculation of evacuation time it is possible to use the simplified method recommended by IMO (MSC.1/Circ.1533) [28], or one of the programs for computer simulation of the evacuation process (discussed in the previous chapter). The optimization method should be chosen for the problem being solved and should take into account the coded input parameters used. The calculated minimum evacuation time should be taken as an objective function, the available evacuation time is a natural limitation. The concept of the available evacuation time is discussed in more detail in the publication [29] and [30]. If the calculated minimum evacuation time exceeds the available time, the determined evacuation plan should be rejected. So the algorithm should look for another solution. It is possible that starting from a different starting point will lead to completely different results. However, if it becomes impossible to find a solution that does not exceed the imposed limitations, it is necessary to modify the arrangement of the evacuation routes. If both, the arrangement of the evacuation routes and the evacuation plan are positively verified, the information obtained can be used at a later stage.

**Fig 1:**
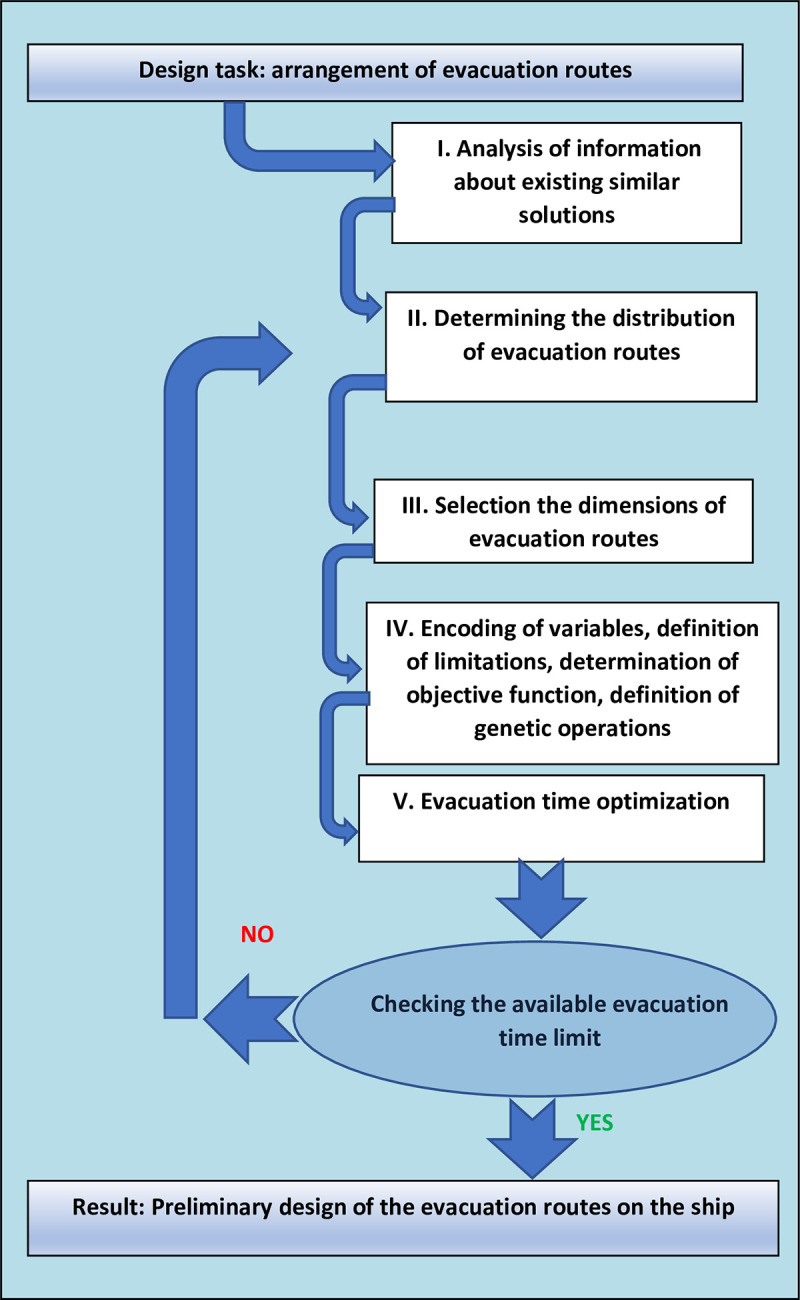
Diagram of the method of evacuation route validation. Source: Own elaboration.

Fig 1 shows the scheme of the algorithm for validating the designed evacuation routes According to the presented algorithm, in the task of designing evacuation routes on the ship, it is necessary to determine their arrangement and dimensions.

The analysis of existing similar solutions allows for their non-random selection. After the initial selection of the arrangement and dimensions of the evacuation routes, they are encoded into a form to be used by the genetic algorithm. The definition of limitations, objective function, and genetic operations is followed by a search for the optimal distribution of human flow along the evacuation routes and a check of the criterion of available evacuation time. Determination of the arrangement and dimensions of evacuation routes based on the presented algorithm allows to obtain variants of evacuation routes for which the evacuation time would be minimal. Special attention should be paid to the use of the method in ship design. Based on the calculations, it can be determined whether the proposed arrangement of evacuation routes is suitable, whether the evacuation routes have sufficient capacity, or whether there is a risk of “bottle necks”.

### Encoding the arrangement of evacuation routes on a passenger ship based on graph theory

An important step is to determine how to code the arrangement of evacuation routes. The first step of the calculation in determining the direction of evacuation for each group of people, regardless of the optimization method adopted, is to code the initial values of the decision variables, and the final step will be to decode the values of the variables coded in the best solution obtained. Thus, determining the method of encoding and decoding the parameters of the objective function is the most important and difficult task to solve. The coding method is one of the main factors that affect the quality of the obtained solutions.

Analyzing how evacuation routes are coded by using tiles representing each room, an analogy to drawing a directed graph is noticed. In relation to the ship’s evacuation plans, the sinks are the vertices representing the initial rooms, while the sources will be the vertices of the assembly stations.

The main advantage of this coding is to determine in which direction passengers will move to get to the assembly stations (movement from one vertex to another). This type of coding does not accurately locate people in a room because the vertices representing the room or corridor do not correspond to the real dimensions. However, in the search for the shortest evacuation time, the choice of this method seems to be the most advantageous, as it will produce sufficiently accurate results without involving very large computational power.

### Calculation of evacuation time for a group of people

In simulation models of evacuation, the movement of people considering evacuation delays can be represented as follows [31]:

- Determination of the speed and flow individually to each person or whole population based on space density,
- Determination of the speed, flow and density values for some spaces by the program user.
- Determination of the minimum specific distance to other people, walls or other obstacles.
- Determination of the capacity—the structure of the model is divided into cells on which passengers move, each cell is assigned a numerical value (the so-called potential affecting the attractiveness of a given road). The evacuation participant follows the potential map and tries to reduce it with each step.
- Determination of the availability of the next cell in the grid—on some models, the passenger may not be able to occupy a cell if it is already occupied by another passenger.
- Determination of conditions—traffic taking place in the structure depends on the existing environmental, structural conditions or the presence of other passengers.
- Determination of passenger traffic based on traffic equations.
- Calculating only uninterrupted passenger flow and improving the result obtained by subtracting or adding delay times.

Selecting the correct form of the function that calculates evacuation time is a nontrivial and very important problem. In most computer models that simulate evacuation, the user assigns a specific speed to the population or to individual participants, given the increased density that can cause congestion and slow traffic in various ways.

When calculating the density-dependent traffic, the calculation method must be selected according to the accepted method of coding the evacuation route structure. The movement of people depends on the environmental and structural conditions that exist along the evacuation routes. The speed of people can be determined from the appropriate equations of motion.

In order to calculate the shortest evacuation time, it seems most advantageous to choose the method of calculating the uninterrupted flow of passengers and to use appropriate coefficients increasing the obtained calculation result, because it will avoid the use of very large computing power and will be suitable for the previously adopted method of coding the layout of the evacuation route based on graph theory [32].

In order to take into account the movement of passengers on evacuation routes when formulating the task of optimizing the evacuation route, the concept of edge weight is introduced. This is the number assigned to the edge. The weight of the edge e can be given by W (e). The path weight e_1_, e_2_,.., e_m_ in graph G is then called the sum of W (e_i_) [33].

**Fig 2:**
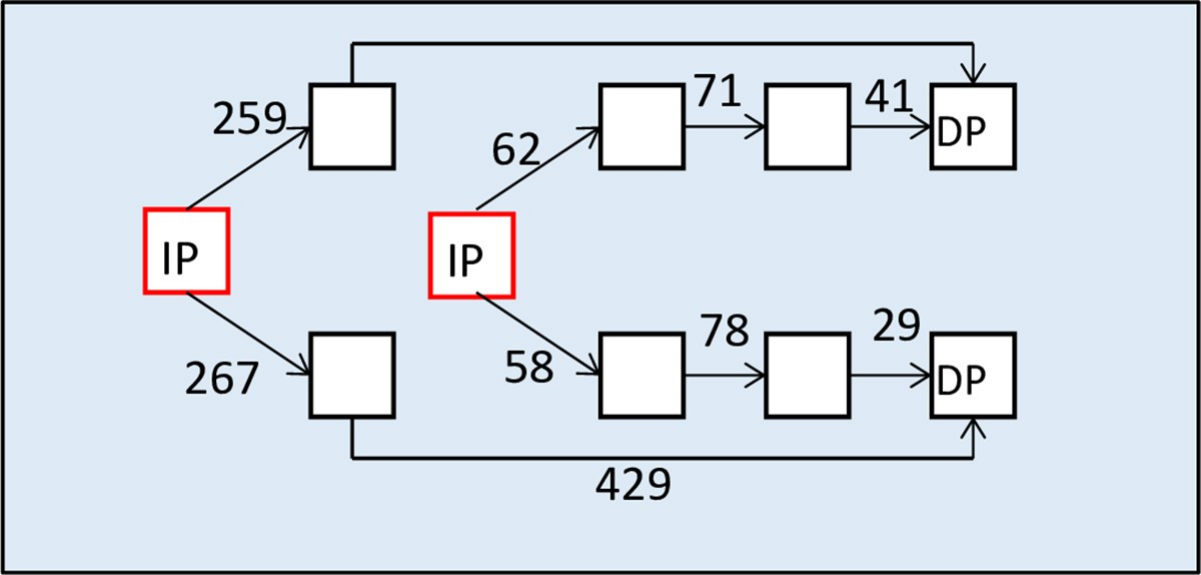
An example of a graph showing the coding of escape routes for a passenger ship. IP-initial points, DP-destination points.

An example of a directed graph with scales showing the travel time [sec] along the escape route section from initial point to destination point is shown in Fig 2.

The squares are the successive points of the escape routes, while the time required to move from one to the next is expressed in seconds, e.g. 259, 267, 429, etc. It has been determined from the base calculations depending on the assumed dimensions of the corridors.

Each edge transition in a directed graph requires some costs. This is the evacuation time needed to cross the edge. In the graph symbolizing the arrangement of evacuation routes on the ship, it is rarely the case that all edges will cost the same (then the cheapest route would be the shortest). Usually there are different clearances of evacuation routes and different levels of difficulty (e.g. stairs), so edge costs will vary.

The smallest weight of the path leading from the source to the sink is called the minimum weight and we denote the symbol W *, while the corresponding path is called the minimum path. In the digraph, which symbolizes the coding of evacuation routes, the weight of a given edge can be interpreted as the time of evacuation of a single evacuation group through this edge.

To calculate the time of evacuation from the ship, it is proposed to use formula 1 [34], used to solve the problem of finding the quickest path. In the graph symbolizing the arrangement of evacuation routes on the ship, the vertices of the graph are given as i_i_. The path P: = (i_1_, i_2_,. . ., i_k_) is given, i.e. a route consisting of individual elements of evacuation routes.

The time it takes for a certain number of people *σ* to move from *i*_*1*_ to *i*_*k*_ along path *P* is:

$$t_{I}(\sigma ,P)=\lambda (P)+\frac{\sigma }{b(P)}$$ (1)

where:

*b(P)*–path capacity, defined as:

$$b(P)=\underset{1\leq i\leq k-1}{\min}b(i_{i},i_{i+1})$$ (2)

(wherein b(i_i_,i_i+1_) is the edge capacity),

and

*λ(P)*—path length (in time), defined as:

$$\lambda (P)=\sum_{i=1}^{i=k-1}\lambda (i_{i},i_{i+1})$$ (3)

(where λ (i_i_,i_i+1_) is the edge lenght).

The goal is to move the assumed number of people from their initial position to their destination as quickly as possible. However, the concept of "fastest" depends not only on the time taken by a single person, but also on the number of people traveling along this path. There is an analogy to the “travel duration” *T* and “flow duration” *t*_*F*_ defined in [28]. Thus, the length of the edge can be saved using the following formula:

$$\lambda (i_{i},i_{i+1})=\frac{L(i_{i},i_{i+1})}{S_{śr}}[s]$$ (4)

where L(i_i_, i_i+1_) is the length (in terms of distance) of a given element of the evacuation route and S_śr_ is the average speed of people moving along the evacuation route.

The width of the edge can be expressed by the formula:

$$b(i_{i},i_{i+1})=Fs(i_{i},i_{i+1})\cdot Wc(i_{i},i_{i+1})$$ (5)

where *Fs* called “specific flow” [person/m·s] is the number of escaping persons past a point in the evacuation route per unit time per unit of clear width *Wc* of the route involved.

Weights t_I_ are assigned to each edge of the graph:

$$t_{I}(i_{i},i_{i+1})=\lambda (i_{i},i_{i+1})+\frac{\sigma }{b(i_{i},i_{i+1})}$$ (6)

The method of determining the speed and flow of people described above is based on the geometry of the analyzed space (density). The method allows calculation of uninterrupted flow. The time obtained should be increased by appropriate factors.

In conclusion, it can be said that the adopted method of calculating the time of people’s movement along evacuation routes using graph theory has the following advantages:

- it is compatible with the adopted method of coding the distribution of escape routes,
- allows to take into account different capacity and different degrees of difficulty of escape routes by using costs of individual edges,
- allows to use one of the methods of searching for minimal graph roads for selecting the shortest (in terms of time) evacuation routes.

The disadvantages of the method adopted include the inaccuracy of the result obtained because of the following assumptions:

- all persons start evacuation at the same time and move in the direction of the designated routes, without overtaking or interaction during movement;
- the speed of persons on particular sections of the route is assumed constant depending on the initial density;
- persons can move unhindered having all routes available in accordance with the evacuation plan;

## Results and discussion

### Searching for the shortest evacuation time by genetic algorithms

The choice of the optimization method was primarily guided by the previously adopted method of coding the distribution of escape routes based on graph theory. Due to the fact that analytical methods of optimization such as e.g. simple gradient method, Newton’s method or golden division method require continuity and differentiability of functions and cannot be applied to discrete problems, they were excluded when solving the problem of optimizing the evacuation layout.

The next group of methods are review methods consisting in reviewing all possibilities and choosing the best one, they are used only for small sets of acceptable solutions, that is, never in real problems.

Limitations of analytical and review methods caused the necessity of searching for other optimization methods. Attention has been drawn to evolutionary methods, which are computer simulations of species evolution process. Their advantage is that they maintain a balance between a broad exploration of the space and the use of previous results, they are random methods, but the search is not conducted "blindly". Algorithms of this type have numerous practical applications

Evolutionary methods allow obtaining good results (unfortunately, the probability of finding an optimal solution is small). It is influenced by the way they work and the randomness of searches. It is not a contraindication in their application, because not always optimization consists in solving a mathematical problem of finding an extreme value of the objective function. Often the search for a conditional extremum of the function is carried out and solutions lying inside the acceptable area of possible solutions are evaluated.

Taking into account the analysis of existing applications of evolutionary methods, their universality and low requirements concerning the form of objective function, it was decided to use them for optimization of evacuation routes layout.

Model of genetic algorithm operation, in which the method of coding input parameters has been adapted to the problem being solved and consists of the following steps.

#### Adoption of a method of coding the parameters of the problem

The parameters of the optimization task are presented in the form of a vector (not necessarily binary) according to the principle of the genetic algorithm. Each of the vectors in the population is a potential solution to the task. The vector consists of *x*_*i*_ positions describing a given feature of the solution. In the considered case, it is assumed that the vector encodes the number of people in a given stream of people following the evacuation route. The vector can be represented in the form of: {x_1_,x_2_,…,x_i_,….,x_p_}.

Consider developing a plan for the fastest evacuation of *N* passengers from _n_ initial rooms *PP [1], PP [2]. . .. . . PP [n].* Passengers have a choice of evacuation routes *P* with different levels of difficulty (stairs, different lengths and widths of corridors) that lead to *k* destination points *DP [1], DP [2]. . . DP [k]*. The number of people in the initial rooms is given *N*_*PP[*__*1*__*]*_, *N*_*PP[*__*2*__*]*_,*…*, *N*_*PP[j]*_,*…*., *N*_*PP[n]*_ and for each initial room, corresponding streams of people *x*_*i*_ are assigned, which leave the rooms. Each evacuation route *P* consists of individual elements of evacuation routes (edges of the graph) defined by their weights (formula 6).

#### Setting the limits

It is necessary to set limits on the number of evacuees in the group to avoid impervious solutions. The limits have been written through formulas 7, 8 and 9.


$$\sum_{i=1}^{p}x_{i}=\sum_{j=1}^{n}N_{PP[j]}=N$$ (7)

$$0\leq x_{N_{PP[j]}}\leq N_{PP[j]}$$ (8)

$$\sum_{i=s}^{t}x_{PP[i]}=N_{PP[j]}$$ (9)

- The first limitation means that the sum of people in all group of people should be equal to the total number of passengers.
- The second limitation specifies the size of the group from initial room. It can be taken in the range from zero to the total number of people in the initial room, but it can also be set at a different level, so that, for example, the distribution of people is more proportional to the width of emergency exits.
- The third limitation imposes that the sum of people in the group coming out of the room should be equal to the total number of people in the room.

#### Determining the objective function

Objective function assesses the "quality" of a given vector in the entire population. On its basis, individuals are selected for further genetic operations. In the problem under consideration, the objective function determines the evacuation time of all passengers. The objective function has a significant impact on the operation of the algorithm, so it is important to define it properly.

Assuming that each edge of the graph representing the arrangement of evacuation routes is assigned weights [*λ*(*i*_*i*_,*i*_*i*+1_), *b*(*i*_*i*_,*i*_*i*+1_)], in addition, there are *s* evacuation routes *P* (paths in the graph), and edge weight t_I_ is calculated according to formula 6, objective function can be expressed by using a following formula:

$$f(x_{1},x_{2},\ldots \ldots ..,x_{p})=max\{t_{I(P1)},t_{P(P2)},\ldots \ldots ..,t_{I(Ps)}\}$$ (10)

Evacuation routes *P* can be taken as parallel and for the further calculations the time of the slowest group of people is used, i.e. the maximum of the obtained travel times for all evacuation routes *max{t*_*I(P1)*_,*t*_*P(P2)*_,*……*..,*t*_*I(Ps)*_*}*. Therefore, an evacuation plan is sought for which the maximum evacuation time occurring on all routes will be minimal. The optimization process minimizes the objective function *f(x*_*1*_, *x*_*2*_,*……*..,*x*_*p*_*)* determined by formula 10.

#### Selection

Selection into the parental population is inspired by natural selection in nature, when the best adapted individuals are more likely to survive. The simplest selection method is the roulette method, in which each vector receives a slice of a roulette wheel proportional to the value of its objective function. Then a random draw is made to the parent population. There is the option of choosing other selection methods such as ranking or tournament methods. Vectors that were selected during the selection are subjected to genetic operations.

#### Genetic operations

Using the genetic operation it is possible to obtain the vectors with new genetic material by crossing and mutation. Multi-point crossing, even crossing or inversion are also available. Using genetic operations, vectors representing new solutions are introduced into the population, contributing to the exploration of new areas of the solution space and searching in the neighborhood of already generated solutions.

#### Replacement of the generation

When the genetic operations are completed, the created vectors partially replace the vectors from the previous generation. In each subsequent iteration, the values of the current population’s objective function are calculated.

#### Calculation examples

In order to verify the method and show that it will be possible to find the shortest evacuation time using a genetic algorithm depending on the arrangement of the evacuation routes, the evacuation time was calculated. The calculations were performed for simple examples of the arrangement of evacuation routes on ships, for which a set of all possible solutions can be created. Having all possible solutions, it is possible to determine what the optimal solution is and whether the genetic algorithm finds it.

**Fig 3:**
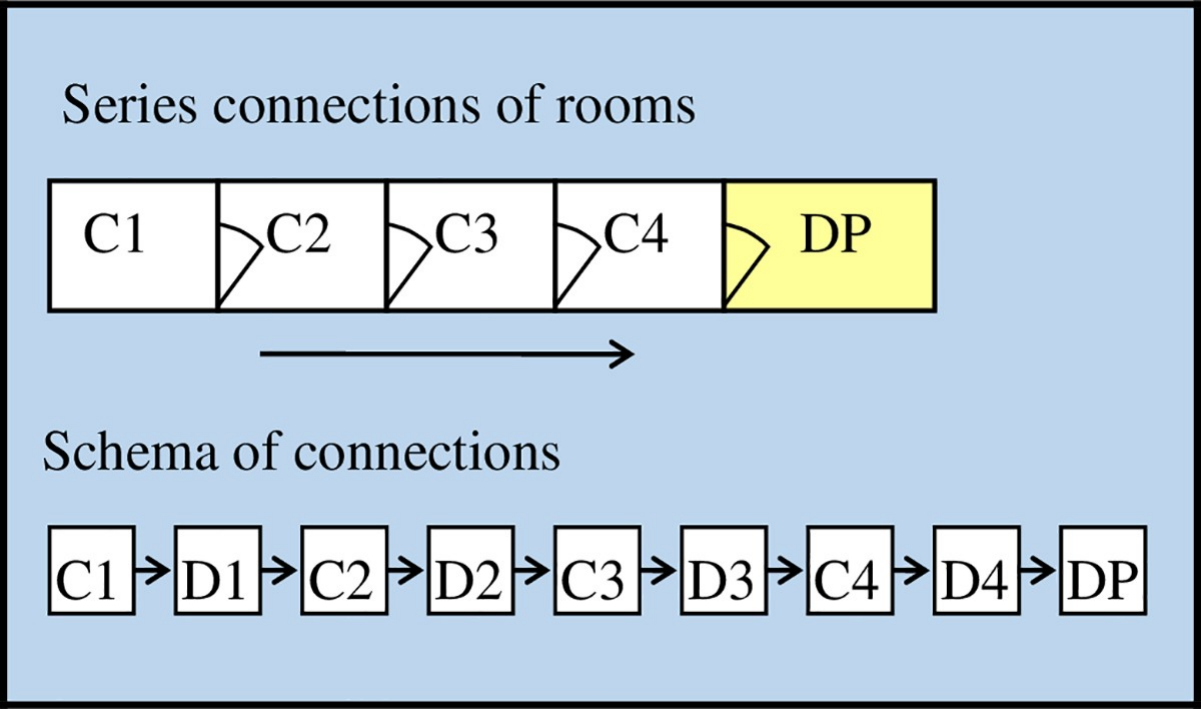
Series connections of rooms and their schema. C- corridors, D- doors, DP-destination point.

*Example 1*. As an example of a simple solution of evacuation routes, serial connection of rooms was assumed, with the movement of passengers from the last room to the first, through subsequent rooms ("walk-through" rooms). In such an arrangement, there is a risk of bottle-neck in the case of different capacity of individual rooms. In the example presented in Fig 3, four people are located in rooms C1, C2, C3, C4 and move to the destination point DP.

Each room in the order C1, C2, C3, C4 is assigned the number 0, 1, 2, 3 or 4 denoting the number of people in the room so that their sum is 4 in each case. The set of all possible configurations is as follows and consists of 35 elements:

Z = {(1111),(0400),(4000),(0040),(0004),(0013),(0031),(0130),(0103),(1003),(1030),

(3001),(3010),(0310),(0301),(3100),(1300),(1102),(1120),(1201),(1210),(2110),(2101),

(0112),(0121),(1012),(1021),(2011),(0211),(2002),(0220), (2020),(0022),(2200),(0202)}

The application of the complicated evacuation time estimation methods presented in [28], is unnecessary in such simple case, which is why a simple method of calculating the flow of people through individual nodes of the system was used [35].

The time t_c_ of k_x_ passengers passing a given vertex (corridor, door) is:

$$t_{c}=\frac{A}{v_{śr}}+\frac{k_{x}}{\left(F_{s}\cdot W_{c}\right)}$$ (11)

where:

*t*_*c*_*−*calculated evacuation time, s

*A*–corridor lenght, m

*v*_*śr*_*−*average speed of people, m/s

*k*_*x*_*−*the number of people passing through the room at any given time

*F*_*s*_*−*specific flow, person/m·s

*W*_*c*_*—*clear width, m

The total evacuation time from the initial vertex to the final vertex is the sum of *t*_*c*_ times calculated for subsequent system vertices. In case of parallel connections, the maximum from the *t*_*c*_ times is selected.

**Fig 4:**
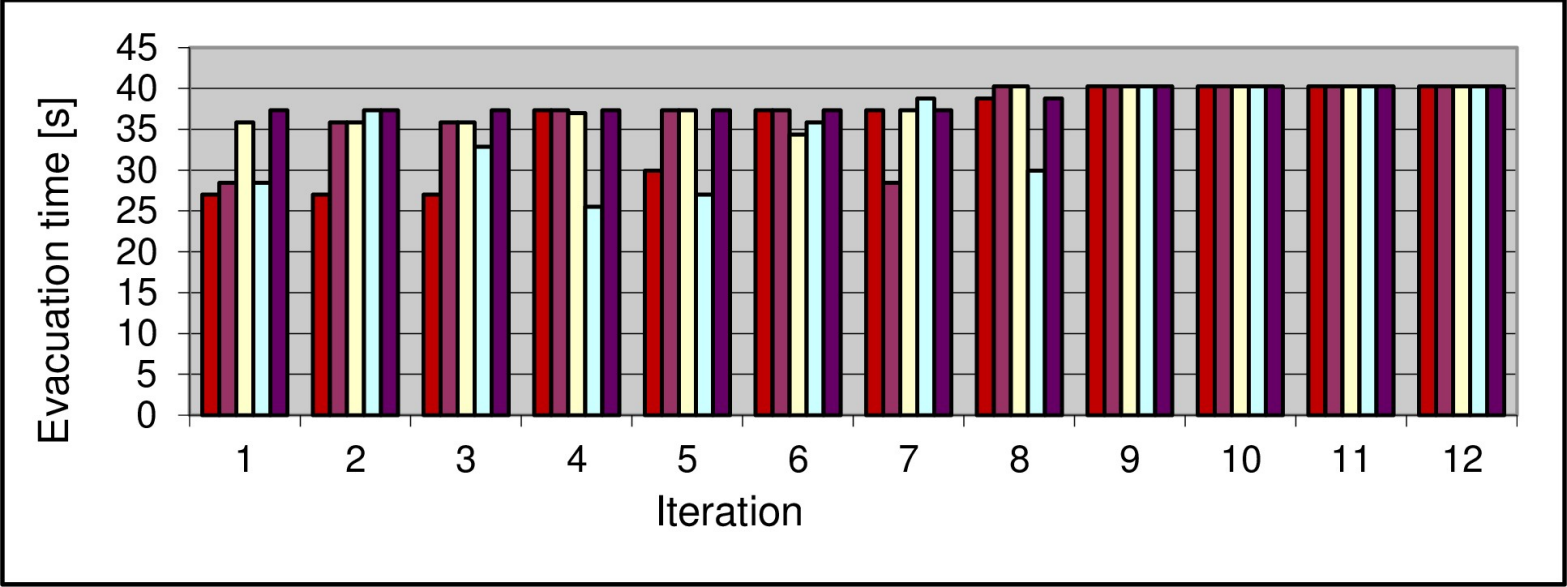
Evacuation time obtained in each iteration using genetic algorithm method.

The initial population consisted of 5 vectors with 4 values. Several trials were made to find the optimal solution using a genetic algorithm. The algorithm found the optimal solution after nine to fifteen iterations (Fig 4).

Each vector is assigned a different color. In successive iterations, the algorithm found better and better solutions as the maximum evacuation time for each vector increased indicating that successive generations were better and better matched.

In the case when 4 people simultaneously start the evacuation from corridor C1, the evacuation time is about 40 seconds and this is the worst result obtained. The minimum evacuation time was achieved for the case when 4 people simultaneously start evacuation from the C4 corridor. The time was reduced by almost half as it was 22 seconds.

**Fig 5:**
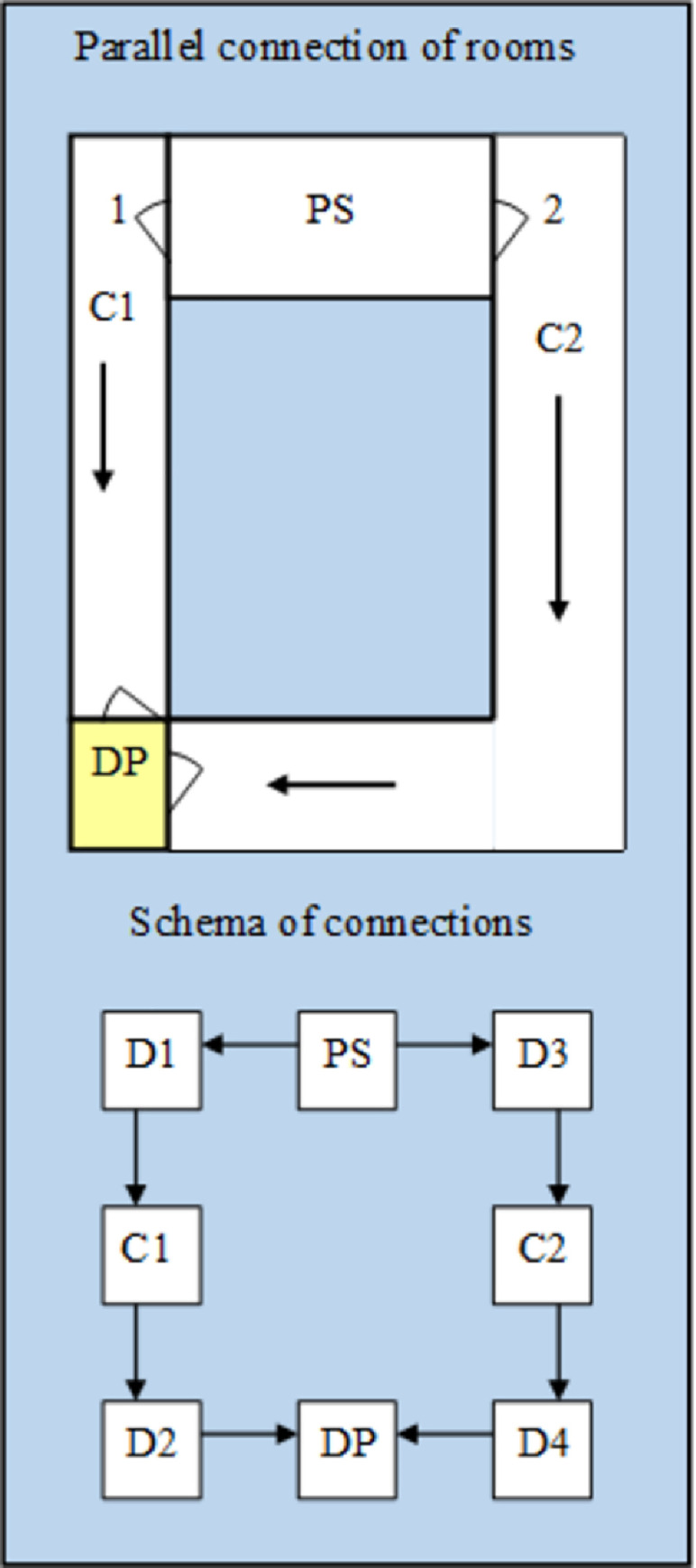
Parallel connection of rooms and their schema. *C- corridors, D- doors, DP-destination point, PS- public space*.

*Example 2*. The parallel connections of the rooms were verified (Fig 5). People evacuate them alternatively depending on the choice of the emergency exit. So the dilemma of choosing the evacuation route appears: wider and longer, or narrower and shorter? Which solution will be better? It definitely depends on the number of evacuees. This example assumes that there are 20 people in the PS room when evacuation begins. They have a choice of two evacuation routes to the destination point. They can follow corridor C1 or corridor C2.

It seems obvious that the evacuation time will be the longest if all people go to the longer and narrower C2 corridor. Below is a set of all possible solutions, including combinations of the number of people choosing a particular emergency exit.

Z = {(0–20),(20–0),(10–10),(19–1),(1–19),(18–2),(2–18),(17–3),(3–17),(16–4),(4–16),

(15–5),(5–15),(14–6),(6–14),(13–7),(7–13),(12–8),(8–12),(11–9),(9–11)}

**Fig 6:**
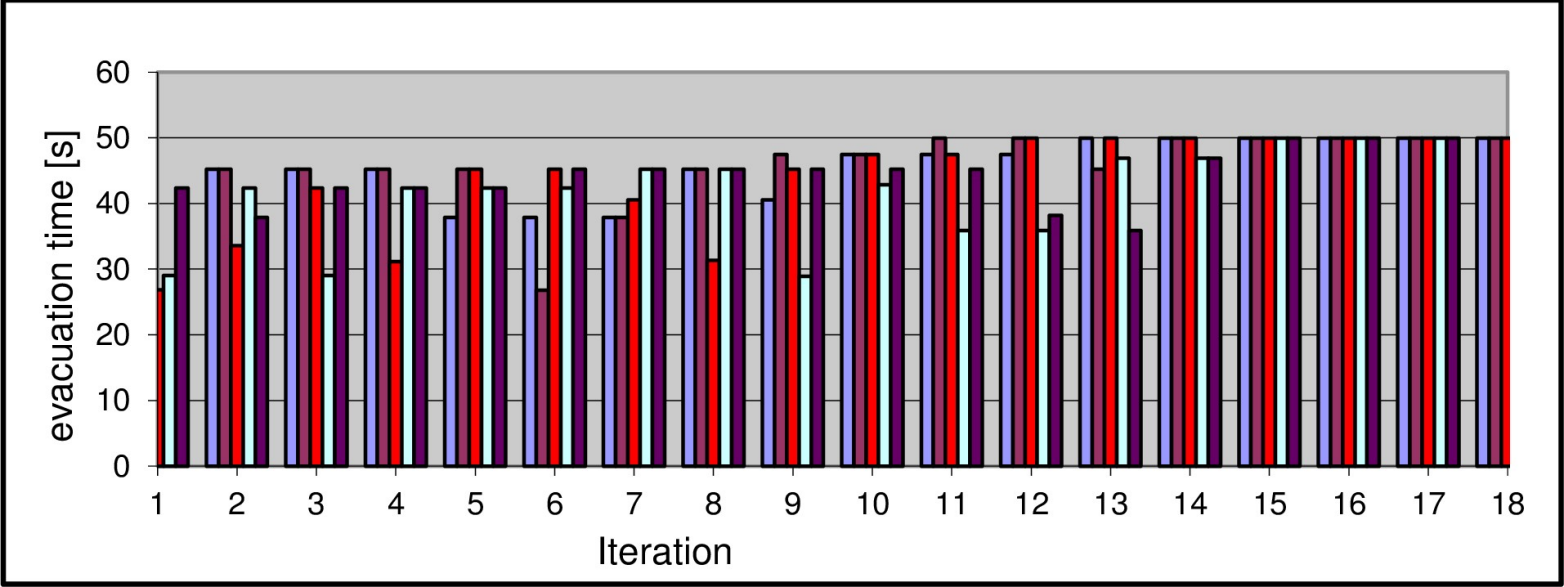
Evacuation time obtained in each iteration using genetic algorithm method.

For the above example, a genetic algorithm was created and several attempts were made to search for the longest evacuation time. In individual tests, the algorithm achieved convergence to the optimal result from the fifteenth to the twentieth generation. Fig 6 shows the results of the simulation.

The population consisted, as in the previous example, of 5 vectors marked with different colors in the graph. Simulation results are presented for the sample in which the algorithm obtained the optimal result in the 15th generation. As in example 1, the graph shows how the results improved during subsequent iterations of the genetic algorithm. If all people go to the assembly point through the C2 corridor, the evacuation time will be 50 seconds. The shortest evacuation time was achieved when 11 people choose the C1 corridor while the other 9 people choose the C2 corridor and it has been shortened to 26 seconds.

### Optimization of evacuation plan on the example of a typical passenger ship

**Fig 7:**
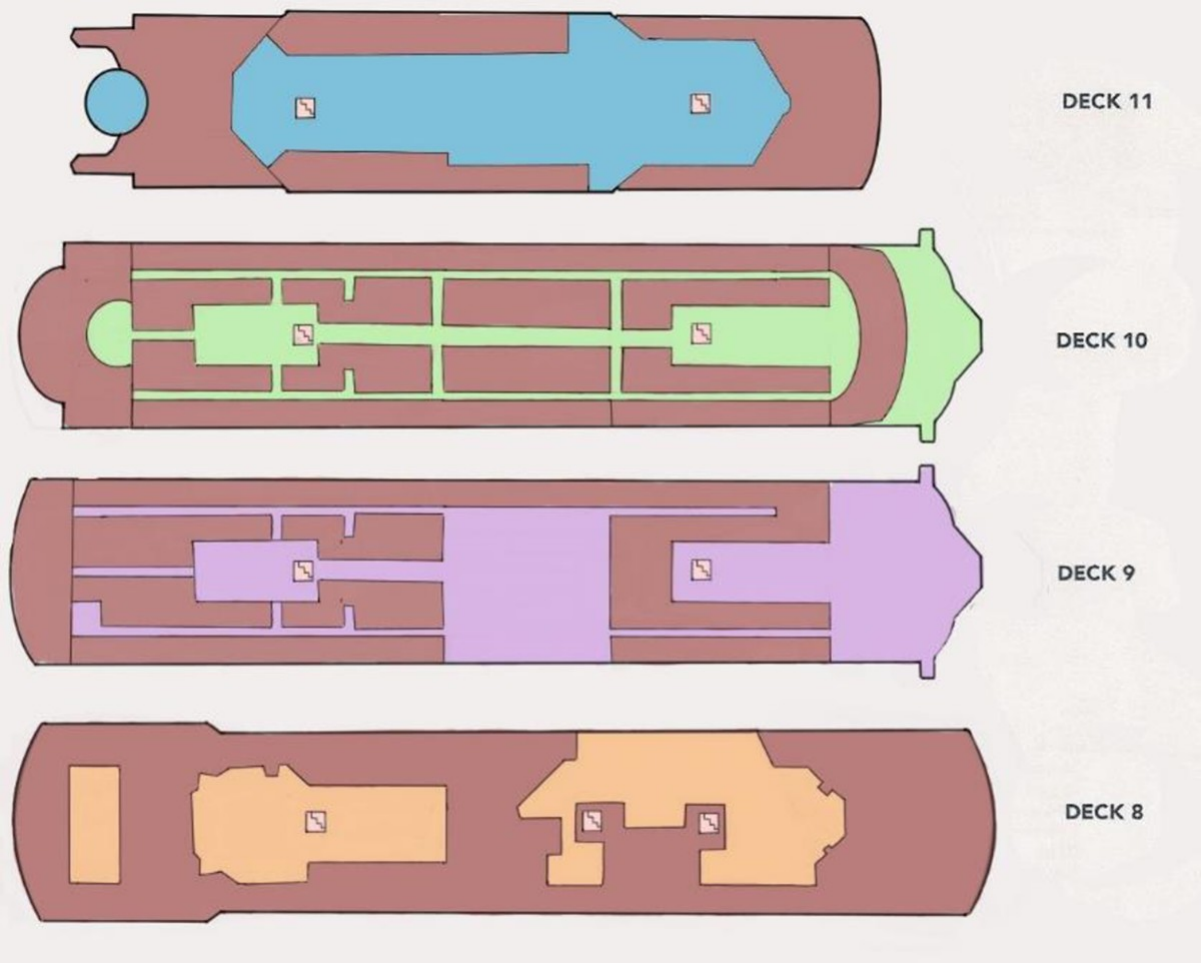
The evacuation route arrangement plan.

**Fig 8:**
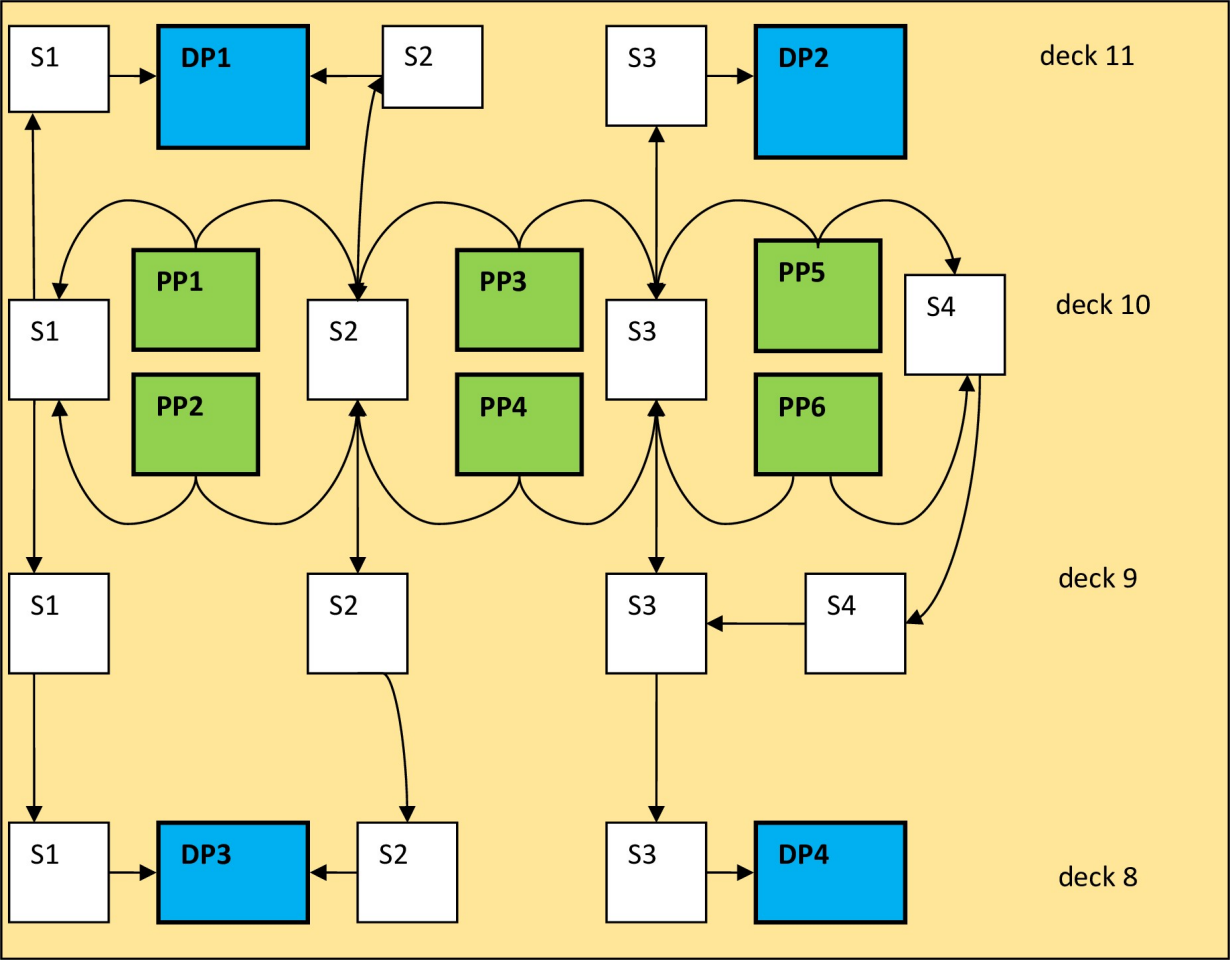
Schema of evacuation routes. (PP- initial, S-staircases, DP- destination points).

The following calculations will be performed for a typical passenger ship with a more complex arrangement of evacuation routes and a much larger number of persons on board. The evacuation route arrangement plan is shown in Fig 7, and a schematic representation of this plan is shown in Fig 8.

In the first stage, calculations were made according to the procedure presented by the IMO in the document MSC.1/Circ. 1533 [28]. The calculations include the case of evacuation of passengers in night conditions, when most of them stay in cabins. Evacuees have the option of going to two assembly stations on deck 11 and two assembly stations on deck 8. They can use four staircases. The total evacuation time of passengers from this ship was 57 minutes, assuming that:

- awareness time for a night evacuation scenario it is 10 minutes,
- the launghing time is 30 minutes

The calculated time for all persons from initial points to reach the appropriate assembly station is 17 minutes. The total time of evacuation does not exceed the assumed standards of the time available for evacuation, which is 60 minutes, however, it was very close to the upper limit.

In the next stage, the data on the analyzed vessel and the verified method were used, and the evacuation plan was optimized.

In order to compare the results obtained by the method of evolutionary algorithms with the results obtained by other methods, functions for optimization available in the Optimization Toolbox of MATLAB were used. The first function used was the nonlinear minimization with constraints (fmincon). The basis of the implementation in the “fmincon” function is the use of Lagrange multipliers. The data for the calculations are entered in M.file containing the objective function and M. file defining the nonlinear inequality and equality constraints of the solved task. For the calculations the objective function written in M.file, which was used to calculate the evacuation time by means of evolutionary algorithms, is used. Other parameters of the algorithm are given in the window of Optimization Toolbox module. Starts were made both from randomly chosen points and by declaring values of variables from the best results obtained by evolutionary algorithm. The minimum evacuation time was 11 minutes (identical results were obtained by the evolutionary algorithm method).

An evacuation time of about 11 minutes was also achieved when using the pattern search method. The name of this method was invented by Hook and Jeeves, while its first applications are attributed to Enrico Fermi and Nicholas Metropolia of Los Alamos National Laboratory. The method belongs to a group of numerical optimization methods that do not require knowledge of the gradient of a function, so it can be used for functions that are not continuous and differentiable. The method works similarly to gradient methods, i.e., it searches for the probable direction of the largest gradient of the objective function (at minimization) and follows this direction with a specified step.

Table 1 summarizes the calculations of evacuation times obtained with the use of genetic algorithms and other mentioned methods.

**Table 1:** Calculation results of the evacuation time of a passenger ship.

Calculation method	Time of evacuation of all persons from the starting points to the appropriate assembly stations [min]	Total time of evacuation from the ship [min]
**MSC.1/Circ. 1533**	17	57
**Genetic algorithm method**	11	46
**Nonlinear minimization with constraints**	11	46
**Pattern search method**	11	46

The time available for the evacuation is 60 minutes, so the time calculated using the method recommended by IMO is within the time allowed. However, it is only slightly shorter than the assumed critical time, after which further evacuation becomes difficult or completely impossible. It should also be remembered that the available time of 60 minutes has been established for cases where the reason for the evacuation is fire. In other cases, it may be even shorter. Evacuation time using the method contained in MSC.1/Circ. 1533 was calculated assuming a certain flow of people during the evacuation scenario. However, during an actual evacuation, a completely different scenario may occur, if only because of an outbreak of panic during the evacuation.

## Conclusions

The problem of passenger ship safety is one that both designers and scientists will have to face in the coming years. The subject proposed in the article has been widely developed in the world recently, this is due to the fact that so far not all hazards associated with sea travel have been eliminated, which can be confirmed, for example, by the spectacular accident of the cruise ship "Costa Concordia". One of the aspects of ensuring the safety of people on board a passenger ship is planning an evacuation strategy in case all possibilities of returning the ship to port have failed.

Improved chances of survival during evacuation from ships are influenced by, among other things: the efficiency of the evacuation, properly designed evacuation routes, knowledge of the layout of rooms and emergency exits, proper interpretation of evacuation signs or quick initiation of evacuation.

The developed method allows to evaluate the arrangement and dimensions of evacuation routes, at the stage of ship design. The novelty and originality of the tools presented in the article, which contribute to the development of maritime transport safety theory, are:

- solving the problem of finding the minimum evacuation time using genetic algorithms, the implementation of which is accompanied by examples of various applications in solving problems related to evacuation problems,
- development of the method of coding the arrangement of evacuation routes as a directed graph with weights, which allows to determine for each route the evacuation time depending on the size of the group of people,
- development of vector coding in a genetic algorithm, formulation of objective and constraint functions so that no incorrect individuals are received during the simulation.

The main advantage of the calculation method adopted was to determine the direction in which passengers would move to reach assembly areas (movement from one vertex to another). This type of coding does not allow the exact location of people inside a room, because the vertex representing the room or corridor does not match the actual dimensions. However, in order to search for the optimal distribution of people along evacuation routes, the choice of this method seems to be the most advantageous, since it will allow to obtain sufficiently accurate results without involving very large computational power. “Course” type networks divide the different floors of a given structure into rooms, corridors, stairs, etc. where passengers move from one of these locations to another. The choice of "course" networks as a coding method allows to use definitions and actions contained in graph theory, their implementation to solve problems related to finding the most advantageous evacuation routes in order to minimize evacuation time. In order to search for the optimal distribution of people along the evacuation routes, the choice of the method of calculating the undisturbed flow of passengers and the use of appropriate augmentation coefficients seems to be the most advantageous, since it will avoid the involvement of very large computing power and will be suitable for the previously adopted method of coding the distribution of evacuation routes based on graph theory.

The calculations showed additional conclusions. In calculating the evacuation time of an example passenger ship in accordance with MSC.1/Circ. 1533, it was assumed that the evacuating groups are distributed equally on individual routes. However, it should be pointed out that this way of evacuation does not allow to obtain the minimum time. A different way of distributing people reduced the evacuation time by about 10 minutes. Determining the appropriate plan for evacuating people in relation to the available evacuation routes can significantly reduce the total evacuation time. This problem is very important when the capacity of evacuation routes is low.

## Supporting information

S1 Fig Coding scheme for escape routes. (PDF)

S1 Table Dimensions of escape routes leading to DP1. (PDF)

S2 Table Dimensions of escape routes leading to DP2. (PDF)

S3 Table Dimensions of escape routes leading to DP3. (PDF)

S4 Table Dimensions of escape routes leading to DP3. (PDF)

S5 Table The values of occupant distribution and initial density D at the start of evacuation and the values of initial specific flow Fs and speed S and calculated flow Fc. (PDF)

S6 Table Calculation of the transition points of the escape routes and the flow Fc and S through these points. (PDF)

S7 Table Individual and group travel times along evacuation routes. (PDF)

S8 Table Calculation t_F_, t_deck_, t_stair_, t_assembly_ and t_I_. (PDF)

S9 Table Edge length *λ*(*i*_*i*_,*i*_*i*+1_) [s]. (PDF)

S10 Table Edge capacity *b*(*i*_*i*_,*i*_*i*+1_) [person/s]. (PDF)

S11 Table Matrix Aeq and vector beq. (PDF)
