# Supplementary figures and images for: The design of the arrangement of evacuation routes on a passenger ship using the method of genetic algorithms
Source: PLoS One. 2021 Aug 9;16(8):e0255993. doi: 10.1371/journal.pone.0255993 (PMC8351972; doi:10.1371/journal.pone.0255993)

S1 Fig 1 Coding scheme for escape routes (PP- initial rooms, S-staircases, DP- assembly points)

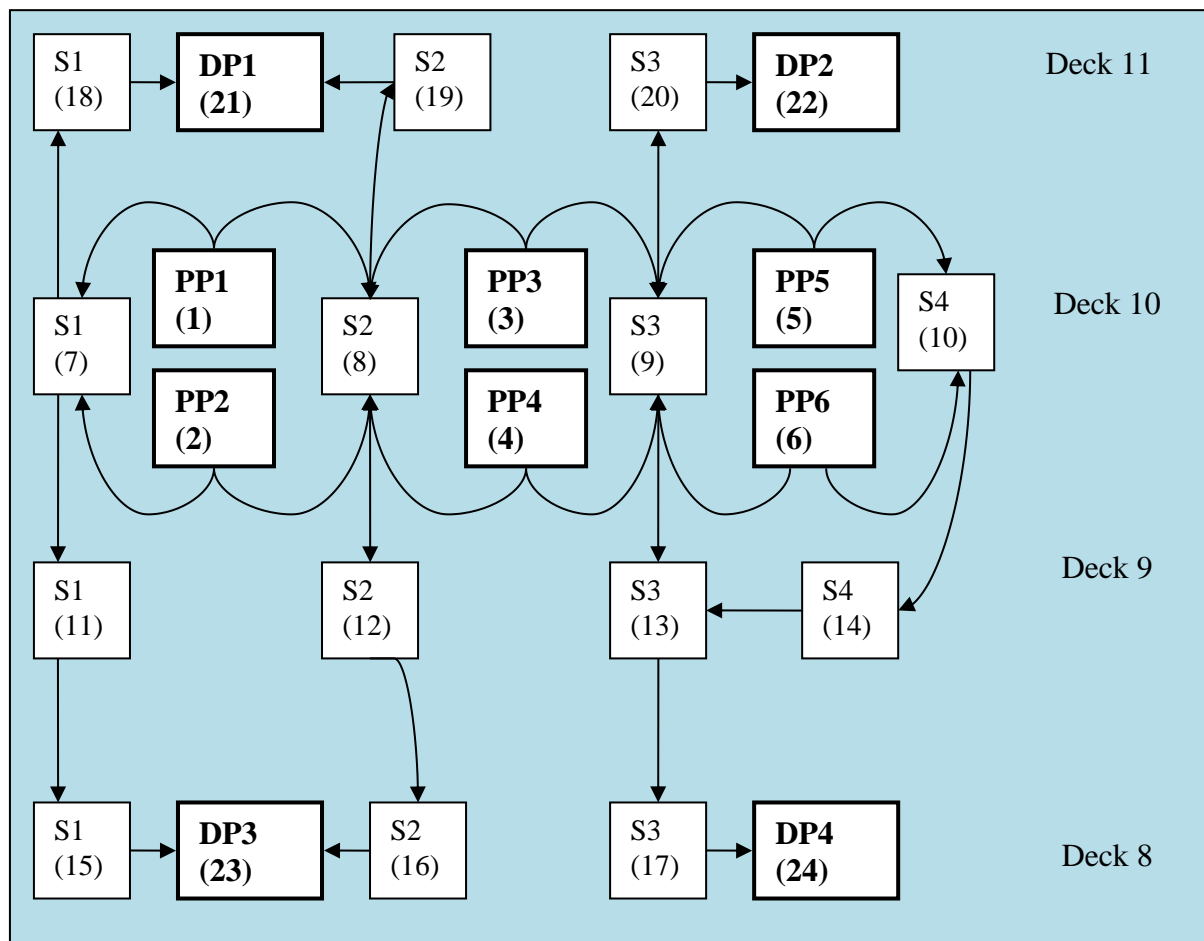

Supplement: S1 Fig — (PDF) [file pone.0255993.s001.pdf]
