# Supplementary material for: The design of the arrangement of evacuation routes on a passenger ship using the method of genetic algorithms
Source: PLoS One. 2021 Aug 9;16(8):e0255993. doi: 10.1371/journal.pone.0255993 (PMC8351972; doi:10.1371/journal.pone.0255993)
Supplement: S1 Table — (PDF) [file pone.0255993.s002.pdf]

S1 Table 1. Dimensions of escape routes leading to DP1

| **Item** | **Width [m]** | **Length [m]** | **Area [m²]** |
|---|---|---|---|
| 1-7 | 1,2 | 9 | 10,8 |
| 7-18 | 1,4 | 6 | 8,4 |
| 18-21 | 6 | 10 | 60 |
| 4-8 | 1,2 | 15,5 | 18,6 |
| 8-19 | 2,4 | 10 | 24 |
| 19-21 | 1,2 | 9 | 10,8 |
| 2-7 | 1,2 | 15 | 18 |
| 2-8 | 1,2 | 14,5 | 17,4 |
| 3-8 | 1,2 | 24 | 28,8 |
| 1-8 | 1,2 | 8,5 | 10,2 |
