# Supplementary material for: The design of the arrangement of evacuation routes on a passenger ship using the method of genetic algorithms
Source: PLoS One. 2021 Aug 9;16(8):e0255993. doi: 10.1371/journal.pone.0255993 (PMC8351972; doi:10.1371/journal.pone.0255993)
Supplement: S2 Table — (PDF) [file pone.0255993.s003.pdf]

S1 Table 2. Dimensions of escape routes leading to DP2

| Item | Width [m] | Length [m] | Area [m²] |
|---|---|---|---|
| 6-9 | 1,2 | 13 | 15,6 |
| 9-20 | 3,2 | 10 | 32 |
| 20-22 | 6 | 10 | 60 |
| 4-9 | 1,2 | 15,5 | 18,6 |
| 5-9 | 1,2 | 13 | 15,6 |
| 3-9 | 1,2 | 15,5 | 18,6 |

S1 Table 4. Dimensions of escape routes leading to DP3

| Item | Width [m] | Length [m] | Area [m²] |
|---|---|---|---|
| 6-9 | 1,2 | 13 | 15,6 |
| 9-13 | 5 | 10 | 50 |
| 13-17 | 5 | 10 | 50 |
| 17-24 | 6 | 25 | 150 |
| 6-10 | 1,2 | 3 | 3,6 |
| 10-14 | 1,25 | 6 | 7,5 |
| 14-13 | 3,6 | 5 | 18 |
| 4-9 | 1,2 | 15,5 | 18,6 |
| 5-9 | 1,2 | 13 | 15,6 |
| 5-10 | 1,2 | 16 | 19,2 |
| 3-9 | 1,2 | 15,5 | 18,6 |
