# Supplementary material for: The design of the arrangement of evacuation routes on a passenger ship using the method of genetic algorithms
Source: PLoS One. 2021 Aug 9;16(8):e0255993. doi: 10.1371/journal.pone.0255993 (PMC8351972; doi:10.1371/journal.pone.0255993)
Supplement: S3 Table — (PDF) [file pone.0255993.s004.pdf]

S1 Table 3. Dimensions of escape routes leading to DP3

| **Item** | **Width [m]** | **Length [m]** | **Area [m²]** |
|---|---|---|---|
| 1-7 | 1,2 | 9 | 10,8 |
| 1-8 | 1,2 | 8,5 | 10,2 |
| 7-11 | 2,25 | 6 | 13,5 |
| 11-15 | 2,25 | 8 | 18 |
| 4-8 | 1,2 | 15,5 | 18,6 |
| 15-23 | 10 | 15 | 150 |
| 8-12 | 3,75 | 10 | 37,5 |
| 2-7 | 1,2 | 15 | 18 |
| 2-8 | 1,2 | 14,5 | 17,4 |
| 3-8 | 1,2 | 24 | 28,8 |
| 12-16 | 3,75 | 10 | 37,5 |
| 16-23 | 3,6 | 15 | 54 |

S1 Table 4. Dimensions of escape routes leading to DP3

| **Item** | **Width [m]** | **Length [m]** | **Area [m²]** |
|---|---|---|---|
| 6-9 | 1,2 | 13 | 15,6 |
| 9-13 | 5 | 10 | 50 |
| 13-17 | 5 | 10 | 50 |
| 17-24 | 6 | 25 | 150 |
| 6-10 | 1,2 | 3 | 3,6 |
| 10-14 | 1,25 | 6 | 7,5 |
| 14-13 | 3,6 | 5 | 18 |
| 4-9 | 1,2 | 15,5 | 18,6 |
| 5-9 | 1,2 | 13 | 15,6 |
| 5-10 | 1,2 | 16 | 19,2 |
| 3-9 | 1,2 | 15,5 | 18,6 |
