# Supplementary material for: The design of the arrangement of evacuation routes on a passenger ship using the method of genetic algorithms
Source: PLoS One. 2021 Aug 9;16(8):e0255993. doi: 10.1371/journal.pone.0255993 (PMC8351972; doi:10.1371/journal.pone.0255993)
Supplement: S4 Table — (PDF) [file pone.0255993.s005.pdf]

S1 Table 4. Dimensions of escape routes leading to DP3

**Item**	**Width [m]**	**Length [m]**	**Area [m²]**
6-9	1,2	13	15,6
9-13	5	10	50
13-17	5	10	50
17-24	6	25	150
6-10	1,2	3	3,6
10-14	1,25	6	7,5
14-13	3,6	5	18
4-9	1,2	15,5	18,6
5-9	1,2	13	15,6
5-10	1,2	16	19,2
3-9	1,2	15,5	18,6
