# Supplementary material for: The design of the arrangement of evacuation routes on a passenger ship using the method of genetic algorithms
Source: PLoS One. 2021 Aug 9;16(8):e0255993. doi: 10.1371/journal.pone.0255993 (PMC8351972; doi:10.1371/journal.pone.0255993)
Supplement: S5 Table — (PDF) [file pone.0255993.s006.pdf]

S1 Table 5. The values of occupant distribution and initial density D at the start of evacuation and the values of initial specific flow Fs and speed S and calculated flow Fc

**Item**	**Person**	**Density [person/m²]**	**Fs [person/(m·s)]**	**Fc [person/s]**	**S [m/s]**
1-7	51	4,7	0,32	0,384	0,1
1-8	51	5	0,32	0,384	0,1
2-7	54	3	0,83	0,996	0,33
2-8	54	3,1	0,77	0,924	0,3
3-8	141	4,9	0,32	0,384	0,1
3-9	141	7,6	0,32	0,384	0,1
4-8	141	7,6	0,32	0,384	0,1
4-9	141	7,6	0,32	0,384	0,1
5-9	117	7,5	0,32	0,384	0,1
5-10	117	6,1	0,32	0,384	0,1
6-9	117	7,5	0,32	0,384	0,1
6-10	117	32,5	0,32	0,384	0,1
