# Supplementary material for: The design of the arrangement of evacuation routes on a passenger ship using the method of genetic algorithms
Source: PLoS One. 2021 Aug 9;16(8):e0255993. doi: 10.1371/journal.pone.0255993 (PMC8351972; doi:10.1371/journal.pone.0255993)
Supplement: S6 Table — (PDF) [file pone.0255993.s007.pdf]

S1 Table 6. Calculation of the transition points of the escape routes and the flow Fc and S through these points.

Item	Persons		Fs in [person/m·s]	Fs max [person/m·s]	Fs [person/m·s]	Fc [person/s]	S [m/s]
	current route	total					
1-7	51	51	0,32	1,3	0,32	0,384	1,2
2-7	54	54	0,83	1,3	0,83	0,996	1,15
7-18	53	53	0,98	0,88	0,88	1,232	0,45
18-21	53	53	0,2	1,3	0,2	1,232	1,2
7-11	52	52	0,61	1,1	0,61	1,37	0,9
11-15	52	52	0,61	1,1	0,61	1,37	0,9
15-23	52	52	0,14	1,3	0,14	1,4	1,2
1-8	51	51	0,32	1,3	0,32	0,384	1,2
2-8	54	54	0,77	1,3	0,77	0,924	1,1
8-19	194	194	0,545	0,88	0,545	1,31	0,75
19-21	194	194	1,1	1,3	1,1	1,32	0,85
8-12	193	193	0,35	1,1	0,35	1,31	1
12-16	193	193	0,35	1,1	0,35	1,31	1
16-23	193	193	0,36	1,3	0,36	1,3	1,2
3-8	141	141	0,32	1,3	0,32	0,384	1,2

4-8	141	141	0,32	1,3	0,32	0,384	1,2
3-9	141	141	0,32	1,3	0,32	0,384	1,2
4-9	141	141	0,32	1,3	0,32	0,384	1,2
9-20	258	258	0,48	0,88	0,48	1,536	0,8
20-22	258	258	0,26	1,3	0,26	1,56	1,2
9-13	258	258	0,31	1,1	0,31	1,55	1
13-17	258	492	0,23	1,1	0,23	1,15	1
17-24	492	492	0,19	1,3	0,19	1,15	1,2
5-9	117	117	0,32	1,3	0,32	0,384	1,2
6-9	117	117	0,32	1,3	0,32	0,384	1,2
5-10	117	117	0,32	1,3	0,32	0,384	1,2
6-10	117	117	0,32	1,3	0,32	0,384	1,2
10-14	234	234	0,61	1,1	0,61	0,76	0,9
14-13	234	234	0,21	1,3	0,21	0,76	1,2
