# Supplementary material for: The design of the arrangement of evacuation routes on a passenger ship using the method of genetic algorithms
Source: PLoS One. 2021 Aug 9;16(8):e0255993. doi: 10.1371/journal.pone.0255993 (PMC8351972; doi:10.1371/journal.pone.0255993)
Supplement: S7 Table — (PDF) [file pone.0255993.s008.pdf]

.

S1 Table 7. Individual and group travel times along evacuation routes

Item	Persons N	Length [m] L	Fc [osoba/s]	S [m/s]	Time t_F [s]	Time t_{deck}, t_{stair} ora $t_{assembly}$ [s]
					t_F=N/Fc	t=L/S
1-7	51	9	0,384	1,2	133	8
2-7	54	15	0,996	1,15	54	13
7-18	53	6	1,232	0,45	43	13
18-21	53	10	1,232	1,2	43	8
7-11	52	6	1,37	0,9	38	7
11-15	52	8	1,37	0,9	38	9
15-23	52	15	1,4	1,2	37	13
1-8	51	8,5	0,384	1,2	133	7
2-8	54	14,5	0,924	1,1	58	13
8-19	194	10	1,31	0,75	148	13
19-21	194	9	1,32	0,85	147	11
8-12	193	10	1,31	1	147	10
12-16	193	10	1,31	1	147	10
16-23	193	15	1,3	1,2	148	13
3-8	141	24	0,384	1,2	367	20
4-8	141	15,5	0,384	1,2	367	13

3-9	141	15,5	0,384	1,2	367	13
4-9	141	15,5	0,384	1,2	367	13
9-20	258	10	1,536	0,8	168	13
20-22	258	10	1,56	1,2	165	8
9-13	258	10	1,55	1	166	10
13-17	492	10	1,15	1	428	10
17-24	492	25	1,15	1,2	428	21
5-9	117	13	0,384	1,2	305	11
6-9	117	13	0,384	1,2	305	11
5-10	117	16	0,384	1,2	305	13
6-10	117	3	0,384	1,2	305	3
10-14	234	6	0,76	0,9	308	7
14-13	234	5	0,76	1,2	308	4
