# Supplementary material for: The design of the arrangement of evacuation routes on a passenger ship using the method of genetic algorithms
Source: PLoS One. 2021 Aug 9;16(8):e0255993. doi: 10.1371/journal.pone.0255993 (PMC8351972; doi:10.1371/journal.pone.0255993)
Supplement: S8 Table — (PDF) [file pone.0255993.s009.pdf]

S1 Table 8 Calculation t_F , t_{deck}, t_{stair}, $t_{assembly}$ and t_I.

**Trasa**	**t_{Fmax} [s]**	**t_{deck}, t_{stair}, $t_{assembly}$ [s]**	**t_I [s]**
1	133	27	160
2	133	35	168
3	148	31	179
4	148	40	188
5	54	34	88
6	54	42	96
7	148	37	185
8	148	46	194
9	367	44	411
10	367	53	420
11	367	34	401
12	428	54	482
13	367	37	404
14	367	46	413
15	367	34	401
16	428	54	482
17	305	22	327
18	428	42	470
19	428	55	483
20	305	32	337
21	428	52	480
22	428	45	473
