# Supplementary material for: The design of the arrangement of evacuation routes on a passenger ship using the method of genetic algorithms
Source: PLoS One. 2021 Aug 9;16(8):e0255993. doi: 10.1371/journal.pone.0255993 (PMC8351972; doi:10.1371/journal.pone.0255993)
Supplement: S9 Table — (PDF) [file pone.0255993.s010.pdf]

S1 Table 9. Edge length $\lambda(i_i, i_{i+1})$[s].

**λ(P)**	**1**	**2**	**3**	**4**	**5**	**6**	**7**	**8**	**9**	**10**	**11**	**12**	**13**	**14**	**15**	**16**	**17**	**18**	**19**	**20**	**21**	**22**	**23**	**24**
**1**							18	17																
**2**							30	29																
**3**								48	31															
**4**								31	31															
**5**									26	32														
**6**									26	6														
**7**											12							12						
**8**												20							20					
**9**													20							20				
**10**														12										
**11**															16									
**12**																20								
**13**																	20							
**14**													10											
**15**																							30	
**16**																							16	
**17**																								50
**18**																					20			
**19**																					18			
**20**																						20		
**21**																								
**22**																								
**23**																								
**24**																								

Źródło: opracowanie własne
