# Supplementary material for: The design of the arrangement of evacuation routes on a passenger ship using the method of genetic algorithms
Source: PLoS One. 2021 Aug 9;16(8):e0255993. doi: 10.1371/journal.pone.0255993 (PMC8351972; doi:10.1371/journal.pone.0255993)
Supplement: S10 Table — (PDF) [file pone.0255993.s011.pdf]

S1 Table 10. Edge capacity $b(i_i, i_{i+1})$ [person/s].

**b(P)**	**1**	**2**	**3**	**4**	**5**	**6**	**7**	**8**	**9**	**10**	**11**	**12**	**13**	**14**	**15**	**16**	**17**	**18**	**19**	**20**	**21**	**22**	**23**	**24**
**1**							1,56	1,56																
**2**							1,56	1,56																
**3**								1,56	1,56															
**4**								1,56	1,56															
**5**									1,56	1,56														
**6**									1,56	1,56														
**7**											1,98							1,58						
**8**												3,3							2,64					
**9**													4,4							3,52				
**10**														1,1										
**11**															1,98									
**12**																3,3								
**13**																	4,4							
**14**													4,68											
**15**																							13	

**16**																							4,68	
**17**																								7,8
**18**																					7,8			
**19**																					1,56			
**20**																						7,8		
**21**																								
**22**																								
**23**																								
**24**																								
